# Process Optimization of the Morphological Properties of Epoxy Resin Molding Compounds Using Response Surface Design

**DOI:** 10.3390/polym16081102

**Published:** 2024-04-16

**Authors:** Julian Vogelwaid, Martin Bayer, Michael Walz, Larysa Kutuzova, Andreas Kandelbauer, Timo Jacob

**Affiliations:** 1 Mobility Electronics, Engineering Technology Polymer & Packaging, Robert Bosch GmbH, 72770 Reutlingen, Germany; martin.bayer2@de.bosch.com (M.B.); michaeldavid.walz@de.bosch.com (M.W.); 2 Fakultät für Naturwissenschaften, Institut für Elektrochemie, Universität Ulm, 89081 Ulm, Germany; timo.jacob@uni-ulm.de; 3 Fakultät für Life Sciences, Reutlingen University, 72762 Reutlingen, Germany; larysa.kutuzova@reutlingen-university.de (L.K.); andreas.kandelbauer@reutlingen-university.de (A.K.); 4 Department of Material Sciences and Process Engineering, Institute of Wood Technology and Renewable Materials, University of Natural Resources and Life Sciences, 1180 Vienna, Austria

**Keywords:** dielectric analysis (DEA), epoxy molding compound (EMC), cure monitoring, glass transition temperature (*T*_g_), design of experiment (DoE), response surface, DiBenedetto equation

## Abstract

An epoxy compound’s polymer structure can be characterized by the glass transition temperature (*T*_g_) which is often seen as the primary morphological characteristic. Determining the *T*_g_ after manufacturing thermoset-molded parts is an important objective in material characterization. To characterize quantitatively the dependence of *T*_g_ on the degree of cure, the DiBenedetto equation is usually used. Monitoring polymer network formation during molding processes is therefore one of the most challenging tasks in polymer processing and can be achieved using dielectric analysis (DEA). In this study, the morphological properties of an epoxy resin-based molding compounds (EMC) were optimized for the molding process using response surface analysis. Processing parameters such as curing temperature, curing time, and injection rate were investigated according to a DoE strategy and analyzed as the main factors affecting *T*_g_ as well as the degree of cure. A new method to measure the *T*_g_ at a certain degree of cure was developed based on warpage analysis. The degree of cure was determined inline via dielectric analysis (DEA) and offline using differential scanning calorimetry (DSC). The results were used as the response in the DoE models. The use of the DiBenedetto equation to refine the response characteristics for a wide range of process parameters has significantly improved the quality of response surface models based on the DoE approach.

## 1. Introduction

A major challenge in the production of thermoset materials such as epoxy resin molding compounds (EMC) is the reliable analysis and optimization of the technological process. Robustness criteria depend primarily on a material’s morphological properties, which are determined by the processing conditions. Monitoring of the process by means of online or inline sensors ensures high product quality and optimal morphological properties of the polymer. The technological window of optimal process parameters can be determined based on response surface plots gained by the design of experiment (DoE) method using response surface analysis. The aim is to obtain mathematical functions for the dependence of final material characteristics on processing conditions and to quantify the effects of individual process factors or their combination based on a limited amount of data.

Epoxy resin molding compounds (EMC) are thermosets with excellent chemical and mechanical properties, good adhesion on printed circuit boards (PCB) or copper, and outstanding electrical insulation properties due to their high content of silicon dioxide particles [1,2]. In the electronics and microelectronics industry for semiconductor devices and microchips, EMCs are ideal as packaging materials for integrated circuit boards, hybrid circuit boards, and transistors [3,4,5,6].

Thermoset processing such as injection, compression, and transfer molding have evolved in recent years in terms of process control and optimization [7]. An important part of molding is the implementation of various sensors in the cavities [8,9]. The data generated by sensors are used for process monitoring and can detect defective parts at an early stage. By monitoring temperature and pressure, process stability can be controlled in terms of cavity filling and reaction temperature [10,11,12]. By analyzing the pressure, the injection process and the filling behavior can be evaluated, and a general indication of the viscosity can be obtained. The decrease in viscosity at a certain temperature is related to the melting process, followed by the initial start of the reaction with the associated increase in viscosity. Therefore, the viscosity during the molding process is significantly influenced by temperature. In addition, temperature triggers the plasticization and the start of the reaction of the material and affects the kinetics of the reaction process [11,13]. Thus, temperature is the most important influencing factor on the formation of a polymer network during the entire transfer molding process [8]. The temperature profile during curing is crucial for the formation of the chemical network structure and, in turn, the morphological properties of thermosetting networks. This was shown, for example, by means of multivariate process trajectories based on spectroscopic data during the curing of solid melamine resin films [14]. Properties of resin samples cured at different temperatures differed substantially and lower curing temperatures could not be compensated for by longer curing times [15]. Monitoring the curing temperature is, hence, indispensable but insufficient since no information about the chemistry of the curing reaction of the molding materials during processing is provided [8,16,17].

In daily practice, it is a huge challenge to optimize the industrial process and ensure consistently high product quality. This is due to the fact that direct process control during the encapsulation process is only feasible via a large number of sensors, and real-time information about the crosslinking state of the material during curing cannot be derived from temperature and pressure sensors [18]. For this reason, the widely used method for determining kinetics is mostly offline and calculated in terms of DSC, DMA, TMA, or rheological methods [19,20,21,22]. The crosslinking conditions, however, are not comparable to those in the cavities of direct packaging processes. Alternative monitoring sensors such as Raman spectroscopy, infrared spectroscopy, ultrasonic monitoring, or dielectric analysis are used to determine the kinetics of the crosslinking process in real time [18,23,24,25,26,27].

Dielectric analysis (DEA) offers great potential for industrial use as a process control instrument as it is robust compared to other sensor technologies, easy to incorporate into tools, and, most importantly, requires significantly less cost [25]. In addition, DEA provides an advantage over optical methods because it is suitable for measuring opaque materials [27,28,29,30]. However, compared to the cure monitoring methods of spectroscopy and ultrasonic monitoring, DEA is dependent on temperature effects, including those caused by shearing [31,32]. Furthermore, spectroscopic methods are more accurate at the beginning of the reaction, during melting, and at the end of the reaction, when ionic motion is already frozen by the existing polymer network [27,30]. With regard to the temperature dependence of the DEA signal, Franieck et al. were able to introduce a temperature compensation factor that eliminates the influence of temperature on the measurement technique and enables standardization of the DEA signal [31].

**Figure 1:**
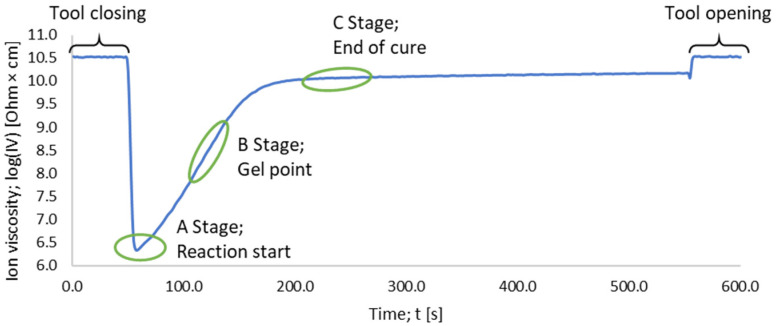
A DEA curve of a measurement at 175 °C is showcased. The three reaction stages: the reaction start A, the gel point B, and the end of cure C are labeled according to [25].

For further understanding, the DEA signal shown in Figure 1 needs to be interpreted at the molecular level. Therefore, the curing state can be described in terms of several stages. Thermosetting materials with crosslinkers can be described by three different stages (i.e., A, B, and C) [25].

During the A-stage, the ions can move freely in the thermosetting material and the resistivity measured via the dielectric analysis is at its minimum. Therefore, a minimum in the ion viscosity is observed. For the A-stage, the degree of cure is considered as zero. Afterwards, in the B-stage, the material starts to react and builds up oligomers. During this B-stage, the ion movement is inhibited due to the formation of small polymer networks. This causes the resistivity to increase. Afterwards, in the C-stage, the material starts to build highly dense polymer networks. For the C-stage, the degree of cure is considered to be one [25,33]. The mobility of ions is most restricted at this stage along the polymerization process and the measured resistivity during the curing process reaches a plateau and its highest value. The individual stages can also be addressed in a DEA curve (for further visualization, see [25,31,32,33]). Achieving the maximum plateau value, however, does not always refer to a complete reaction. Materials with a high glass transition temperature (*T*_g_), where processing takes place below the *T*_g_, will reach the glassy state where *T*_g_ = *T*_Processing_ [33]. The current state of the literature shows a correlation of ionic viscosity with the degree of cure and the *T*_g_ [29,32,33]. However, the degrees of cure obtained by DSC and DEA could not be interrelated properly due to the lack of comparability of the heating rates, leading to different curing behaviors [31,34].

The curing of thermosets is characterized by two independent phenomena: gelation and vitrification [35]. Gelation corresponds to the initial stage of polymer network formation with an infinite average molecular weight, while vitrification is associated with the transition from a rubbery state to a glassy state. Polymerization can be described by a time–temperature–transition (TTT) phase diagram in which the transformation depends on temperature [36]. The relationship between the glass transition temperature *T*_g_ and degree of cure of thermosets is essential and explained by the DiBenedetto equation [35,37,38]. DiBenedetto outlines the relationship between the degree of cure and the *T*_g_ using a bended linear curve that can be described by the following equation [35,37]:

(1) $$\frac{\left(T_{g}-T_{g0}\right)}{\left(T_{g1}-T_{g0}\right)}=\frac{\lambda \;\alpha }{\left(1-\left(1-\lambda \right)\alpha \right)}$$

*T*_g0_ is the glass transition temperature of the raw material in an uncured state, whereas *T*_g1_ is the glass transition temperature of the fully cured material. *λ* is a material fitting constant and influences the shape of the DiBenedetto curve. *T*_g0_ is determined during the initial heating and before the reaction begins. The *T*_g_ determined during heating is not only a function of the rate of temperature rise, but rather of thermal history. The highest independence of the *T*_g_ from the heating rate is only achieved if the heating rate is high enough, e.g., 10–20 K/min to minimize post-crosslinking. High heating rates lead to a shift of the *T*_g_ to a much higher value due to the heat distribution. Consequently, the heating rate must be reduced to minimize the *T*_g_ shift, whereas the effect of post-crosslinking is enhanced. Therefore, the determination of the *T*_g_ at a certain degree of cure after a certain crosslinking process is a major challenge when evaluating the thermomechanical properties of the material [35,37,38]. Conventional *T*_g_ determination methods such as differential scanning calorimetry (DSC), dynamic mechanical analysis (DMA), or thermo mechanical analysis (TMA) themselves increase the *T*_g_ during the characterization process due to the slow heating process generating post-crosslinking. Regarding process optimization, the development of new methods for determining the degree of cure at a certain degree of cure after different process steps are urgently required to correlate the degree of cure to the *T*_g_.

Several studies have demonstrated a correlation between ionic viscosity and the degree of cure [25,31,32,33,34,39]. Franieck et al. showed a linear relationship between the ionic viscosity and *T*_g_ by introducing a temperature compensation factor [31]. Furthermore, attempts have been made to apply the DiBenedetto equation to ionic viscosity. Assuming that the ionic viscosity correlates with the *T*_g_, the DiBenedetto equation can be applied to the ionic viscosity [40,41]. In this work, a new method to determine the *T*_g_ at a certain degree of cure is presented. In addition, a validation of the application of the material specific fit value *λ* to the course of the ionic viscosity values over the degree of cure is demonstrated. A design of experiment (DoE) approach is used to demonstrate the comparability of DEA and DSC and their correlation to the *T*_g_.

## 2. Materials and Methods

### 2.1. Materials

**Figure 2:**
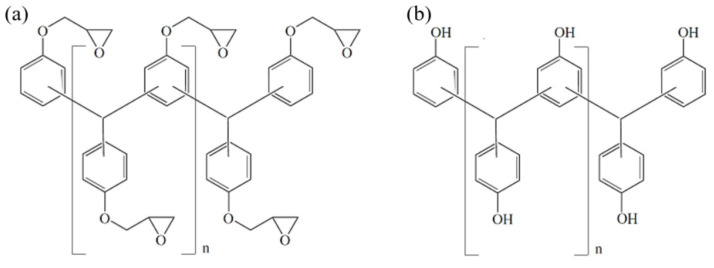
Multifunctional epoxy resin (**a**) and multifunctional phenolic hardener (**b**).

A commercially available pre-mixed epoxy mold compound (EMC) with a high filler content (83 wt% spherical silica particles) and with a nucleophilic curing agent was used in this study. The basic chemical structure of a multifunctional epoxy resin is presented in Figure 2a and for a multifunctional phenol hardener in Figure 2b. The material is received as pellets. The material was stored at 2 °C and warmed up to room temperature for >8 h prior use.

The material is ideal for insulating printed circuit boards, e.g., in the mobility sector. Here, high glass transition temperatures and electrical insulation are required, which is achieved by epoxy–phenolic compounds processing at high temperatures and a high proportion of silica particles. In addition, the rapid reaction at temperatures between 165 °C and 185 °C of the epoxy–phenolic reactive resin allows a fast processing time.

### 2.2. Dielectric Analysis (DEA) in Transfermold

**Figure 3:**
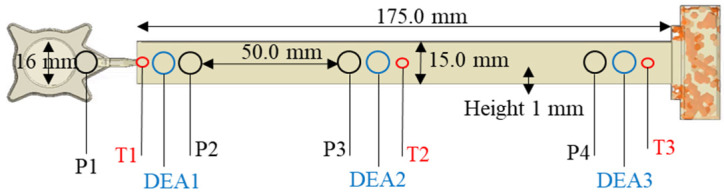
Design and location of the inline sensors in the processing equipment. Thermocouple type K, near the gate (T1) and monotrode for dielectric analysis (DEA1), and near the end of the cavity (T3) and monotrode for dielectric analysis (DEA3).

The dielectric measurements were performed with a 4/3RC monotrode (NETZSCH-Gerätebau GmbH, Selb, Germany) and a temperature sensor thermocouple type K (Kistler Instrumente AG, Winterthur, Switzerland), which were connected to a DEA analyzer (DEA 288 Epsilon, NETZSCH-Gerätebau GmbH, Selb, Germany). The sensors were integrated into a slit-die cavity mold (175.0 × 15.0 × 1.0 mm) mounted on a transfer mold press. The locations of the DEA and temperature sensors inside the mold’s slit-die cavity are shown in Figure 3.

The experiments were carried out using 10 and 100 Hz as the measurement frequencies, which were found to be the frequencies with the lowest noise and the highest reproducibility [16,31]. First, the reproducibility of the DEA signal was checked with kinetic analysis experiments, which were evaluated by performing 6 isothermal inline measurements at different temperatures (165, 175, and 185 °C) and at different injection speeds (1.0, 2.5, and 4.0 mm/s) with a recording time of 5 min.

**Figure 4:**
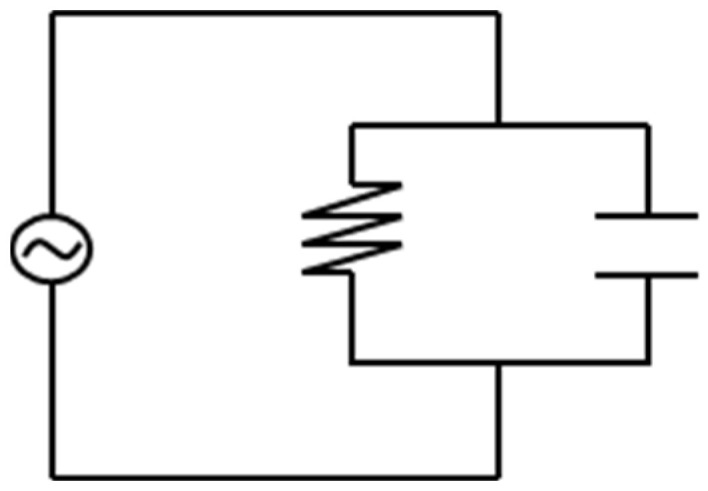
A regular circuit is shown with both an alternating current (AC) and a direct current (DC).

The principle of a dielectric measurement is to apply a conductivity to a material resulting in a measured resistivity. For polymers, the measured conductivity can be described by the contributors for alternating current (AC) and direct current (DC) in a regular circuit as shown in Figure 4 [25].

Both contributors can be expressed individually by their correlation as:

(2) $$\rho_{DC}-ion\;movement,$$

(3) $$\rho_{AC}-rotating\;dipoles.$$

For crosslinked polymers, it has been shown that the ion movement (ion viscosity(*IV*)) is the more reliable value to gain insights into the degree of cure [25], compared to the rotating dipoles. Therefore, the resistivity can be expressed by

(4) $$\rho_{DC}=IV=\frac{1}{\sigma_{DC}}=\frac{1}{q\;\mu \;n}$$

where $\sigma_{DC}$ is the time-alternating conductivity (ohm^−1^·cm^−1^), *q* is the magnitude of electronic charge (coulombs), *μ*(*t*) is the free ion mobility (cm^2^/(V·s)), and *n* is the free ion concentration (cm^−3^) [25]. The value for the free ion movement is connected to the Stokes–Einstein equation, which expresses the resistivity as

(5) $$\rho_{DC}=IV=\frac{k_{B}}{q^{2}n\;D_{0}}\cdot T\;e^{\frac{Q}{k_{B}T}}$$

where $D_{0}$ is the diffusion coefficient (cm^2^/s), *k*_B_ the Boltzmann’s constant (eV/K), *T* is the absolute temperature (K), and Q is the heat quantity.

By applying the natural logarithm, the DEA signal can be expressed as:

(6) $${log}_{10}\left(\rho_{DC}\right)=\log_{10}\left(IV\right)=\log_{10}\left(\frac{k_{B}}{q^{2}\;n\;D_{0}}\right)+\log_{10}T+\frac{E_{a}}{k_{B}\;T\;ln\left(10\right)}$$

Data pre-preparation is required to use the ion viscosity values independently. Previous work has shown that a temperature adjustment is required to eliminate the temperature influence on the ion motion [31]. Therefore, two temperature coefficients are applied to be able to compare the values of the ion viscosity between measurements at different temperatures [32].

(7) $${log}_{10}{\left(\rho_{10}\right)}_{norm}=A+\log_{10}\left(T_{norm}\right)+T_{measured}\log_{10}(\rho_{DC})\;+\left(c_{1}T_{measured}+c_{2}\right)\frac{1}{T_{norm}}$$

Alpha can be gained by taking the maximum and minimum values for the ion viscosity and applying them as the values for the minimum and maximum degree of cure [42,43].

(8) $$IV_{max}={\alpha_{IV}}_{max}=1;IV_{min}={\alpha_{IV}}_{min}=0$$

Therefore, the cure index obtained from the ion viscosity $\alpha_{IV}$ can be described as

(9) $$\alpha_{IV}=\frac{\left(IV-{IV}_{0}\right)}{\left({IV}_{1}-{IV}_{0}\right)}$$

Considering the relationship between degree of cure and glass transition temperature by DiBenedetto (Equation (1)), the equation can be transferred to

(10) $$T_{g}=T_{g0}+\frac{\left(IV-{IV}_{0}\right)}{\left({IV}_{1}-{IV}_{0}\right)}\;(T_{g1}-T_{g0})$$

Combining Equations (9) and (10) the following relationship is obtained:

(11) $$\frac{\left(IV-{IV}_{0}\right)}{\left({IV}_{1}-{IV}_{0}\right)}=\frac{\left(T_{g}-T_{g0}\right)}{\left(T_{g1}-T_{g0}\right)}$$

Based on this correlation of the ion viscosity to the glass transition temperature (*T*_g_), a DiBenedetto fit was used to calculate and validate the degree of cure via DEA according to Equation (12) utilizing the same λ fitting constant calculated via the $T_{g}$ measurements.

(12) $$\frac{\left(IV-{IV}_{0}\right)}{\left({IV}_{1}-{IV}_{0}\right)}=\frac{\lambda \;\alpha_{IV}}{\left(1-\left(1-\lambda \right)\alpha_{IV}\right)}$$

The cure index was determined based on the maximum measured ion viscosity value of the epoxy material in preliminary experiments with heating times of 60 min, which was assumed to be 100% crosslinked. The zero-percentage degree of cure was extrapolated using the DiBenedetto fit with the *λ* fitting constant of the material obtained from the glass transition temperature (*T*_g_) analysis, as explained in Section 2.5.

### 2.3. Design of Experiment—Face-Centered Design

**Figure 5:**
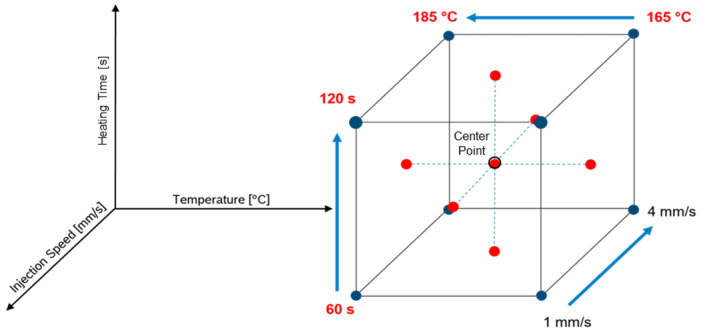
All experimental parameters are shown in a full factorial represented as a face-centered design of experiment (FCDoE).

A face-centered design of experiment (FCDoE) was performed to analyze the process window of the material and to investigate the different effects of the process parameters of temperature, injection speed, and heating time. Correlations to the degree of curing, calculated via the DEA, were determined via the DSC residual enthalpy and to the glass transition temperature, which are referred to as responses. Furthermore, prediction models of all responses were calculated by statistical evaluation of the design of experiment (DoE) data. The detailed factor level settings are summarized in Section 3.4 and the design space covered by the FCD is illustrated in Figure 5.

The center point (CP) represents the experiment performed at overall intermediate factor level settings for all investigated factors within the studied design space. The heating time differs systematically by 30 s (s) and temperature 10 °C towards the center point. The injection speed limits were chosen to be between 1.0 mm/s and 4.0 mm/s. The limits of the factor level settings were defined based on the processability of the material. All DoE data were analyzed using Design Experts software (Stat-Ease, Inc., Minneapolis, MN, USA, Version 8.1.0)

### 2.4. Residual Enthalpy Measurements via Differential Scanning Calorimetry (DSC)

The differential scanning colorimetry (DSC) DSC 204F1 Phoenix^®^ (NETZSCH-Gerätebau GmbH, Selb, Germany) with an integrated auto-sampler was used to calculate the residual enthalpy. All measurements were conducted under a nitrogen atmosphere with a N_2_ flow rate of 40 mL/min. For each measurement, approximately 20.2 ± 0.6 mg was cut from the cured samples near the DEA1 sensor and weighed in aluminum crucibles (Concavus pan and lid made of Al, NETZSCH-Gerätebau GmbH, Selb, Germany), which were sealed and exposed to a temperature ramp ranging from 20 to 260 °C with a heating rate of 20 °C/min. The changes in enthalpy were recorded and analyzed using the Proteus Thermal Analysis software (NETZSCH-Gerätebau GmbH, Selb, Germany, Version 7.1.0). The percentage degree of cure was determined based on the maximum measured enthalpy of the delivered material according to Equation (13) and the measured residual enthalpy of the produced parts. The baseline was used as the zero dimension for each determination of the exothermic areas.

The degree of cure can also be defined by a linear behavior as

(13) $$\alpha_{H}=1-\frac{{\Delta H}_{t}}{{\Delta H}_{total}}$$

According to Equation (13), the degree of cure (*α_H_*) directly correlates with the measured heat flow (∆*H*) during the reaction of the raw material, where *α*_t_ represents the degree of cure at a given time, ∆*H*_t_ is the overall released heat at a specific time, and ∆*H*_total_ corresponds to the overall possible released heat during the complete reaction. Due to pre-crosslinking of the material in its delivery state from the supplier, $\alpha_{H}$ obtained from the residual enthalpy had to be calculated to correct the states of the degree of cure according to the DiBenedetto equation (Equation (1)).

### 2.5. Determination of the Glass Transition Temperature via Warpage Analysis

**Figure 6:**
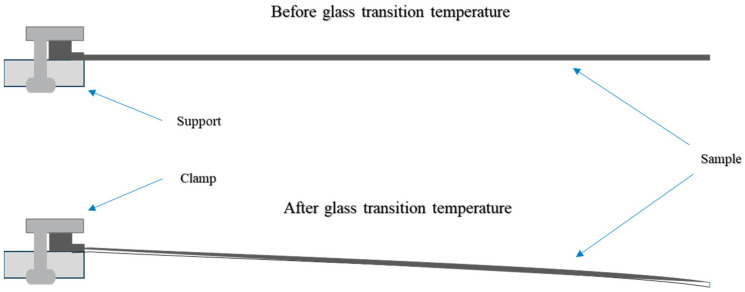
Sketch of set-up for determining the *T*_g_ via gravity bending during heating of a sample in the warpage analyzer.

The principle of the warpage analysis is based on the thermal material expansion. The expansions in *x*, *y*, and *z* directions are measured during a specific heating rate. The scheme of the measurement is illustrated in Figure 6.

**Figure A1:**
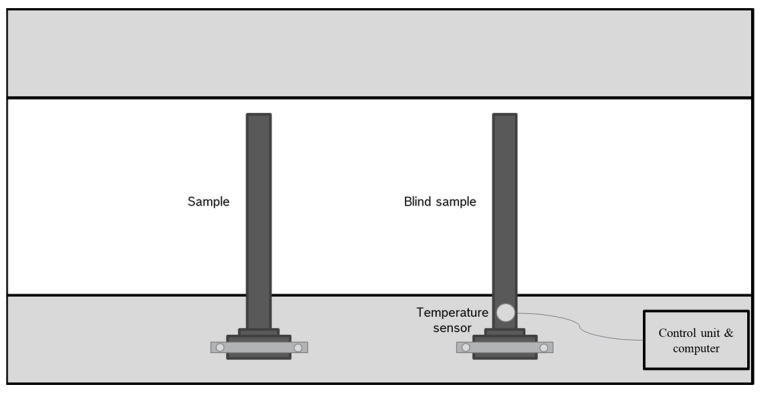
Schematic representation of the measuring setup of the warpage analyzer for detecting the x-value. On the left, the sample is clamped to enable bending via gravity during heating of the material. On the right, a reference sample is clamped including a temperature sensor to detect the actual temperature of the sample.

Gravity was used to determine the *T*_g_ of the samples. The *T*_g_ was identified with the onset and an additional ongoing of the bending of the sample. The samples were clamped from one side with a fixture analogous to a single point bending test and heated between 60 °C and 250 °C with 6 K/min. A reference sample with a temperature sensor was used to measure the accurate temperature within the material without influencing the bending behavior. A sketch of the set-up is shown in Figure A1. The obtained *T*_g_ values were evaluated against the conversion according to the DiBenedetto equation (Equation (1)). By evaluating the *T*_g_ values, the fitting constant *λ* of the material could be determined using Equation (1). The corresponding degree of cure was determined using the residual enthalpies in DSC runs for the samples. *T*_g0_ was set to a value of −15 °C based on DSC measurements and on manufacturer’s calibration classification. Using the resulting DiBenedetto fit, the current degree of cure of the actual state of the material could be calculated. Therefore, the fitting constant *λ* was applied using the DiBenedetto equation of the ion viscosity to Equation (12) to recalculate the degree of cure and to extrapolate the minimum ion viscosity.

## 3. Results and Discussion

**Figure A2:**
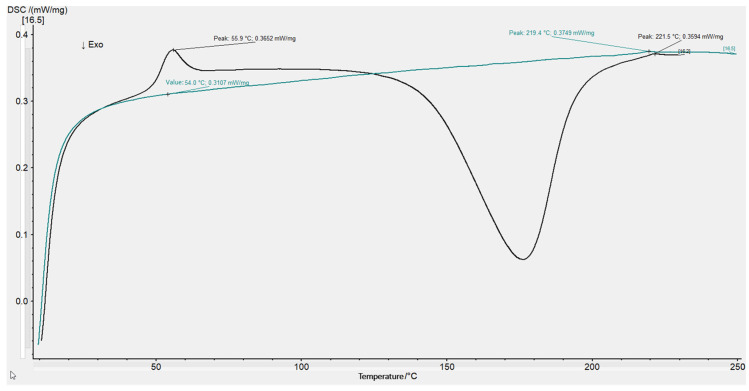
Determination of the *T*_g_ using DSC. First heat-up is used to determine *T*_g_ of the uncured material (black line), second heat-up to calculate *T*_g_ at 100 percent crosslinked material (green line).

All experiments were carried out based on factor level settings given by a full factorial face-centered design of experiment (FCDoE) with variation of the factors of temperature, injection speed, and heating time in the mold tool to investigate the design space more precisely. The limits of the FCDoE were set based on empirical values investigated in preliminary experiment, which focused primarily on the ability to demold the test specimens. The focus of this work is on short heating times of 60 seconds (s), as here the effects of the process factors show the most significant effects. Representative response surface plots for the heating times 90 s and 120 s are given in Figure A2 and Figure A8, respectively. Based on the observed correlation between the ionic viscosity and the *T*_g_, the DiBenedetto equation was applied to compare the target values of glass transition temperature (*T*_g_) and ionic viscosity (*IV*).

### 3.1. Correlation between Ion Viscosity and Glass Transition Temperature

**Figure 7:**
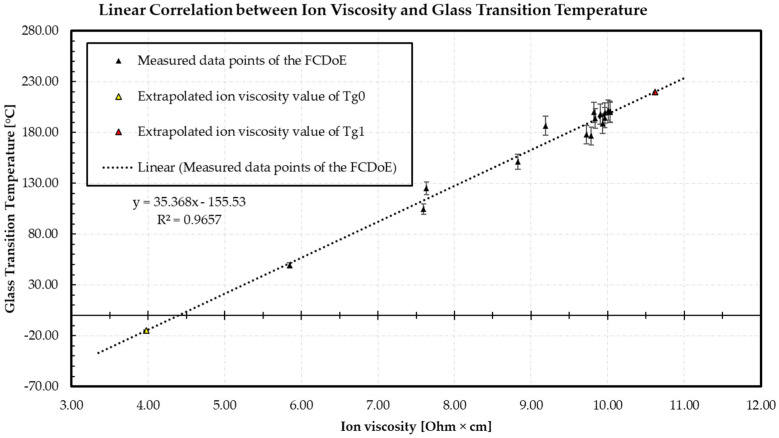
The measuring points of the FCDoE of the dielectric analysis (DEA) respective the ion viscosity are plotted over the measured *T*_g_ values, representing a linear relationship in this range of crosslinking with a regression of 0.9657.

Figure 7 shows a linear relationship between ionic viscosity and the measured *T*_g_ values immediately after molding using the warpage analysis. 

The lowest measured value of 5.99 Ohm·cm shows the minimum ion viscosity at the DEA sensor 1 and is assigned to a *T*_g_ value of 49.1 °C of the raw material measured via DSC. The averaged standard deviation of the ionic viscosity is 0.02 Ohm·cm and of *T*_g_ determinations is 3.5 °C. Using the linear equation, the ion viscosity value of *T*_g1_ (220 °C) and *T*_g0_ (−15 °C), measured by DSC, can be calculated. This results in an ion viscosity value of 10.62 Ohm·cm for *T*_g1_ and 3.97 Ohm·cm for *T*_g0_. The analysis of the ion viscosity and the *T*_g_ values thus confirms the correlation reported in the literature and enables the DiBenedetto equation to be extended to the ion viscosity. Since the raw material is already pre-crosslinked, the DiBenedetto equation can be used to determine the degree of cure.

### 3.2. DiBenedetto—Glass Transition Temperature vs. Conversion

**Figure 8:**
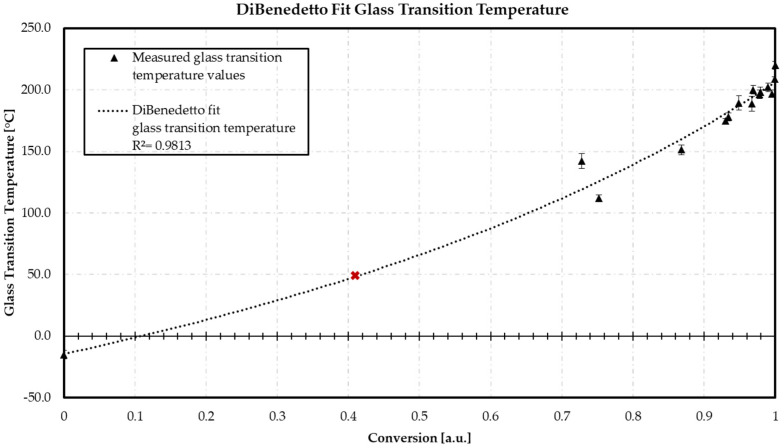
DiBenedetto fit utilizing for the calculation of λ and the conversion of the already precured present material. Red point: measured *T*_g_ of the present unmolded material by DSC. Conversion represents the calculated values via residual enthalpy via DSC.

The following diagram in Figure 8 shows the relationship between the conversion and the glass transition temperature (*T*_g_). The value *λ* is calculated using the DiBenedetto equation and adopted as the fitting constant.

Using the DiBenedetto equation, the *λ* value of the material is determined using the minimum *T*_g0_ in conversion state 0 and the maximum *T*_g1_ in a fully cured state 1. The *T*_g_ results of the warpage analysis are included to calculate the prediction accuracy of the DiBenedetto fit. The regression of the fit has an *R*^2^ of 0.9813. The calculated *λ* value is 0.57. The obtained equation is used to calculate the crosslinking state of the material at the stage of demolding. The average *T*_g_ value of 49.1 °C, measured by DSC for the present material, is shown as a red data point. Inserting the value of 49.1 °C into the generated DiBenedetto fit equation, a conversion of 0.41 is calculated. This results in a current material that is already 41.0% crosslinked.

The calculation of the current degree of cure is used to calculate the measured ion viscosity using the appropriate DiBenedetto equation.

### 3.3. DiBenedetto—Ion Viscosity vs. Conversion

**Figure 9:**
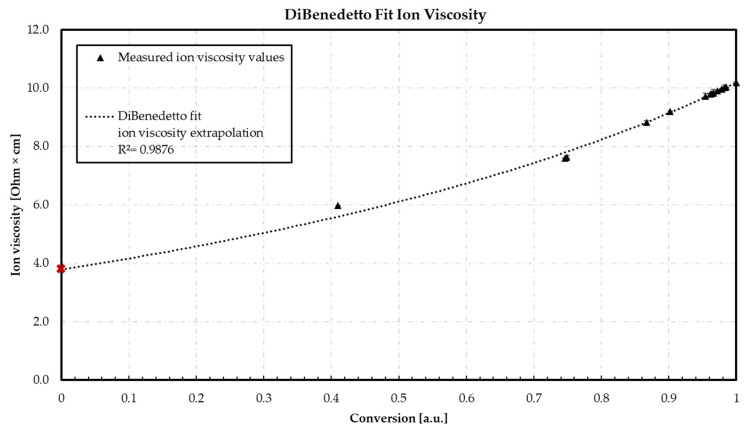
DiBenedetto fit utilizing for the calculation of the minima ion viscosity *IV*_0_. Red point: measured *T*_g_ of the present unmolded material by DSC. Conversion represents the calculated values for *IV*.

The following graph in Figure 9 illustrates the relationship between the conversion and the ion viscosity. The fitting constant *λ* = 0.57 is used to generate a DiBenedetto fit. The potential minimum *IV* is extrapolated based on the DiBenedetto fit: The maximum *IV* is set to the maximum ion viscosity measured in preliminary experiments. The measured values are included to validate the DiBenedetto fit.

The lowest measured value of ionic viscosity of 5.99 Ohm·cm based on the fit shows a predicted conversion of 0.475 at sensor 1. The assumption of a pre-crosslinking of 41.0% based on the DiBenedetto calculation of the *T*_g_ can therefore be considered correct. The difference of 6.5% can be explained by the process conditions due to a reaction already taking place inside the plunger up to sensor 1 during the injection phase. Based on the predicted fit values and the measured ion viscosity value, an accuracy of *R*^2^ of 0.9876 is calculated. Using the fit equation, a minimum ion viscosity value of 3.84 Ohm·cm is predicted via the extrapolation. The deviation of the extrapolated value of the ion viscosity gained by the linear relationship between *T*_g_ and ion viscosity described in Section 3.1 shows only a slight deviation of about 0.13 Ohm·cm. This deviation can be explained by the lower regression of the linear adjustment (*R^2^* = 0.9657) and the associated higher coefficient of variance of *T*_g_ determination compared to the ion viscosity measurements. Therefore, the calculation via the DiBenedetto equation enables a more accurate prediction of the *IV*_0_. In addition, the slight deviations show that the DiBenedetto equation can be used to calculate the degree of cure using the DEA. The resulting degrees of cure are used to evaluate the face-centered design of experiment.

**Figure 10:**
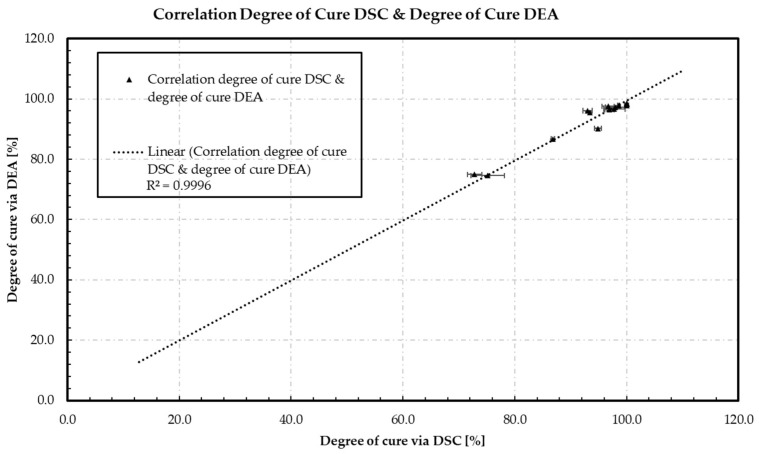
The measuring points of the FCDoE of the calculated degree of cure via dielectric analysis (DEA) obtained from the DiBenedetto approach are plotted over the calculated degree of cure via DSC obtained from the residual enthalpy, representing a strong linear relationship with a regression of 0.9996.

Comparison of the DiBenedetto fit models and the accuracy obtained from the validation with the measured data leads to the assumption that lambda can be used for the calculation of the degree of cure via ion viscosity. As a result, *IV* demonstrates an equal behavior to *T*_g_ according to DiBenedetto, and enables the comparison of the degree of cure calculated via DEA and the degree of cure calculated via DSC. To verify the comparability of the DEA and DSC values obtained, Figure 10 shows the measured data points of the FCDoE of the calculated degree of cure via dielectric analysis (DEA) by DEA versus the calculated degree of cure via DSC.

Figure 10 shows a strictly linear behavior between the calculated degrees of cure of the DEA and DSC methods. Representing an average standard deviation of 1.5%, the data obtained indicate no significant differences comparing both methods. In addition, the DEA method demonstrates lower standard deviations than the DSC method. The DSC method shows significantly higher standard deviations within a lower degree of cure range of 70–80%. In addition, at calculated high degrees of cure of 98% using DEA, no residual enthalpy is detected using DSC, resulting in a 100% calculated degree of cure. A possible explanation is that the sensitivity of the DSC is limited by the low organic content, and non-crosslinked functional groups react during the heating phase of the DSC without generating an exothermic signal. As a result, DEA provides greater accuracy and is more suitable for determining the degree of cure of epoxy molding compounds (EMC). In addition, the extremely low standard deviation of the DEA measurements makes it ideal for in situ and online monitoring. However, it should be emphasized that the degree of cure $\alpha_{IV}$ measured by the DEA cannot be compared with that measured by the DSC ($\alpha_{H})$ without validating the calculation with the ion viscosity of the uncured material (*IV*_0_).

### 3.4. Results of the Design of Experiment (DoE)

First, the measured values of the responses were plotted on the corresponding factor settings, as shown in Table 1. An analysis of variances (ANOVA) was performed to determine the significant effects and to generate the response surfaces. In the main text, to compare the responses, the model prediction of the significant effect between temperature (A) and heating time (C) is discussed as it is the only significant interaction for all responses.

#### 3.4.1. Response Surface of Degree of Cure Calculated via Dielectric Analysis (DEA)

The analysis of variances (ANOVA) used to calculate significant effects of the parameters and their interactions on the responses is shown in Table 2. Based on the results, the response surface is calculated and subsequently discussed.

The model’s F-value of 741.31 means that the model is significant, and there is only a 0.01% chance that such a large F-value could occur due to noise. *p*-values of less than 0.0500 mean that the model terms A (temperature), C (heating time), AC, A^2^, and C^2^ are significant. The F-value for the lack of fit of 10.05 indicates that the lack of fit is significant. The explanation for the high lack of fit F-value relates to the very low variance of the measured data points. The predicted R^2^ of 0.9848 is in good agreement with the adjusted R^2^ of 0.9886, i.e., the difference is less than 0.2. The adequate (adeq) precision measures the signal-to-noise ratio, which at 59.472 is greater than 4, indicating an adequate signal.

**Figure 11:**
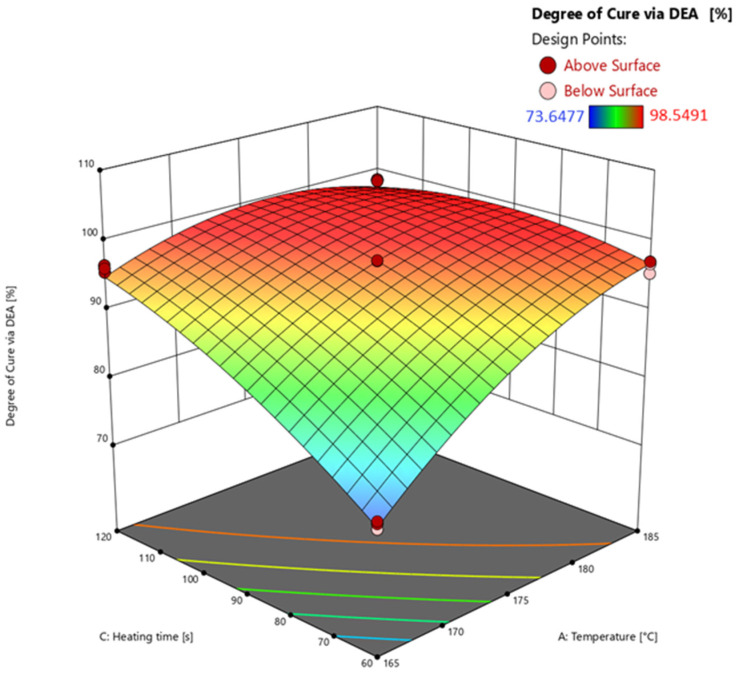
Response surface of the degree of cure calculated via DEA extrapolation. Effects of transfer molding parameters temperature (A) and heating time (C) on the degree of cure (*z*-axis) are plotted. Dark red and light red dots depict the measured raw values above and below the prediction surface, respectively.

**Figure A3:**
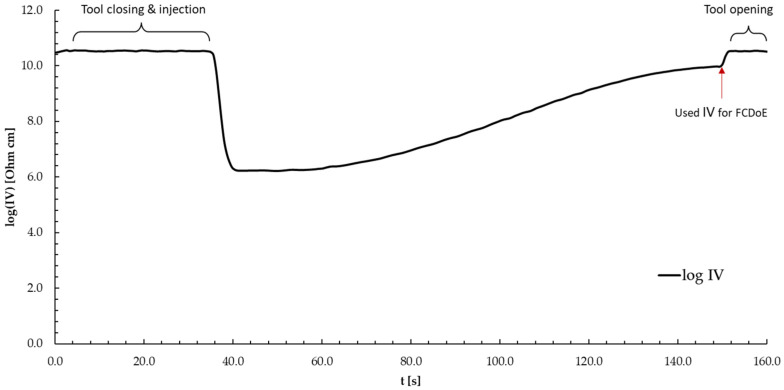
Example of a raw DEA signal of the DoE trial at a tool temperature of 175 °C, injection speed of 2.5 mm/s, and 120 s heating time. The end (red arrow) is used as the maximum reached ion viscosity before tool opening.

The effects of the temperature and heating time on the reaction area of the FCDoE determined by DEA (*z*-axis) can be observed in Figure 11 at an injection speed of 1 mm/s. An example of the DEA measurement of a test point and the determination of the ion viscosity is shown in Figure A3.

Looking at the response surface illustrates the significant effects of temperature (A) and heating time (C) on the calculated degree of cure via DEA. Overall, the higher the temperature and the longer the heating time, the higher the degree of cure. For example, considering axis A at 60 s, the degree of cure increases between 165 °C and 185 °C with an average calculated degree of cure of 96%. The increase due to temperature can be explained by kinetics of the EMC using the Arrhenius equation. The temperature at the exponent significantly determines the kinetics and consequently the course of the reaction. The effect of the heating time on the degree of cure is well illustrated by the C axis at 165 °C. The longer the heating time, the higher the resulting degree of cure. The significant interaction between A and C (AC) can be seen on the middle axis of the diagram. The effect on the degree of cure rises with increasing temperature and simultaneously with increasing heating time. Furthermore, this effect can also be explained by the kinetics of the material. The square significant effects A^2^ and C^2^ result from the general kinetic curve reaching a plateau towards 100% degree of the cure. In addition, A^2^ represents lower measured ionic viscosity (*IV*) values in the plateau at 185 °C compared to the maximum measured *IV* at 175 °C. As a result, the temperature exhibits a quadratic behavior. Therefore, a quadratic response surface equation is obtained for modeling, which calculates the predictions of the degree of cure, e.g., at 185 °C and 120 s, degressively.

#### 3.4.2. Degree of Cure via Differential Scanning Calorimetry (DSC)

Based on the results of the analysis of variances (ANOVA) listed in Table 3, the response surface is calculated and subsequently discussed.

The model’s F-value of 226.44 implies that the model is significant. *p*-values of less than 0.0500 mean that the model terms A, B, AC, A^2^ are significant. The F-value for the lack of fit of 7.57 means that the lack of fit is significant. The explanation for the high lack of fit F-value relates to the very low variance of the measured data points. The predicted R^2^ of 0.9425 is in good agreement with the adjusted R^2^ of 0.9568, i.e., the difference is less than 0.2. The adequate (adeq) precision measures the signal-to-noise ratio, which at 32.894 is greater than 4, indicating an adequate signal.

**Figure 12:**
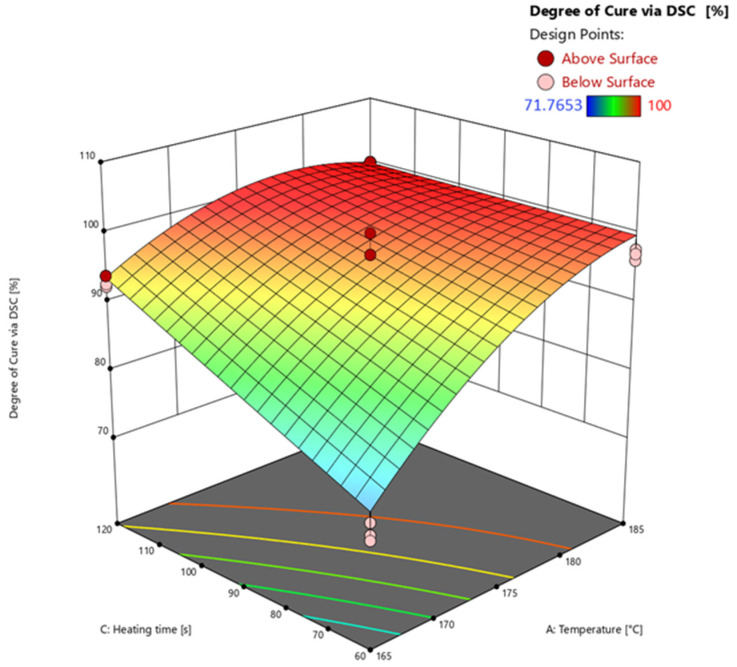
Response surface of the degree of cure calculated via DSC extrapolation. Effects of transfer molding parameters temperature (A) and heating time (C) on the degree of cure (*z*-axis) are plotted. Dark red and light red dots depict the measured raw values above and below the prediction surface, respectively.

**Figure A4:**
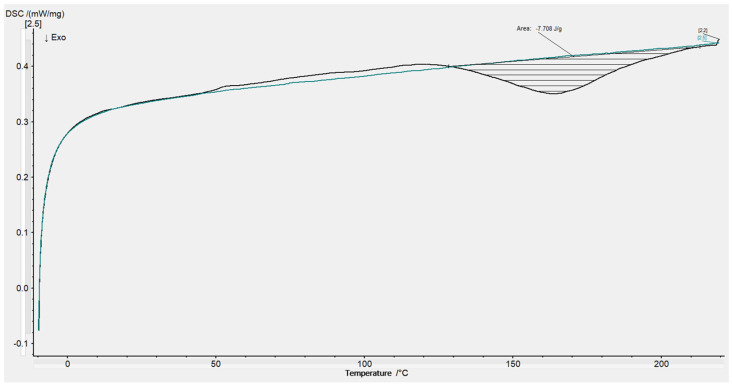
Determination of the residual enthalpy using the first heat up as data line (black line) and the second heat-up data as baseline (green line). Residual enthalpy was calculated via exothermic area analysis and set in proportion to the maximal measured enthalpy of the raw material.

The effects of the temperature and heating time on the reaction area of the FCDoE determined by DSC (*z*-axis) are shown in Figure 12 at an injection speed of 1 mm/s. An example of the DSC measurement of a test point and the determination of the residual enthalpy is shown in Figure A4.

Looking at the response surface shows the significant effects of temperature (A) and heating time (C) on the calculated degree of cure via DSC. As can be seen by looking at the response surface of the degree of cure calculated via DEA, the higher the temperature and the longer the heating time, the higher the degree of cure. For example, looking at axis *A* at 60 s, the degree of cure increases between 165 °C and 185 °C at a calculated degree of cure of 99% on average. The increase due to temperature and heating time can also be explained by kinetics of the EMC using the Arrhenius equation. The time and the temperature significantly determine the kinetics and thus the course of the reaction. The effect of the heating time on the degree of cure is well illustrated by the C axis at 165 °C. The longer the heating time, the higher the resulting degree of cure. The significant interaction between A and C (AC) is recognizable at the center axis of the diagram. The effect on the degree of cure rises with increasing temperature and simultaneously with increasing heating time. The square significant effect A^2^ results from the general kinetic curve reaching a plateau towards 100% degree of the cure. The significant effect of C^2^ compared to DEA, which was not determined according to the ANOVA, results from the measurement method. The residual enthalpy is determined up to a temperature of 260 °C until the end of the reaction, while the degree of cure in the DEA is exclusively determined at the respective set temperatures. As a result, the DEA reaches a plateau and thus a quadratic course. Furthermore, at higher degrees of cure, the DSC is unable to determine the residual enthalpy. As mentioned above, the sensitivity reaches the limits of its capability at these levels. Any possible residual cure reacts during the heating phase without producing a measurable signal. In addition, higher degrees of cure are usually calculated via the DSC. In addition, when using DSC to determine the glass transition temperature (*T*_g_), this process leads to a shift in the *T*_g_ range at higher values.

#### 3.4.3. Glass Transition Temperature via Warpage Analyzer

The results of the analysis of variances (ANOVA) are listed in Table 4. The response surface is calculated and subsequently discussed using the significant effects.

The model’s F-value of 83.02 implies that the model is significant. *p*-values of less than 0.0500 mean that the model terms A, B, C, AB, AC, BC, A^2^, B^2^, and C^2^ are significant. The F-value for the lack of fit of 19.31 implies a significant lack of fit. The explanation for the high lack of fit F-value is the same as discussed above. The predicted R^2^ of 0.9848 is in good agreement with the adjusted R^2^ of 0.9886, i.e., the difference is less than 0.2. The adequate (adeq) precision measures the signal-to-noise ratio, which at 59.472 is greater than 4, indicating an adequate signal.

**Figure 13:**
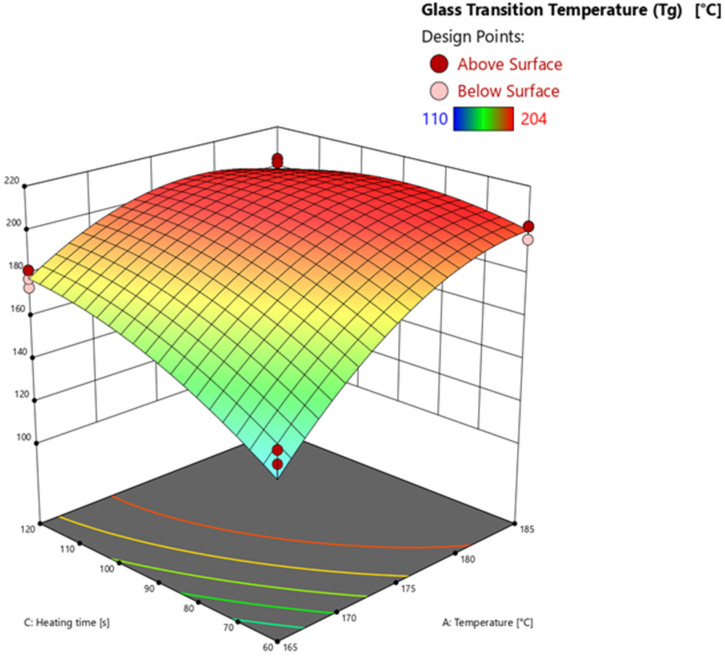
Response surface of the glass transition temperature calculated via warpage analysis shown at an injection speed of 1 mm/s. Effects of transfer molding parameters temperature (A) and heating time (C) on the degree of cure (*z*-axis) are plotted. Dark red and light red dots depict the measured raw values above and below the prediction surface, respectively.

**Figure A5:**
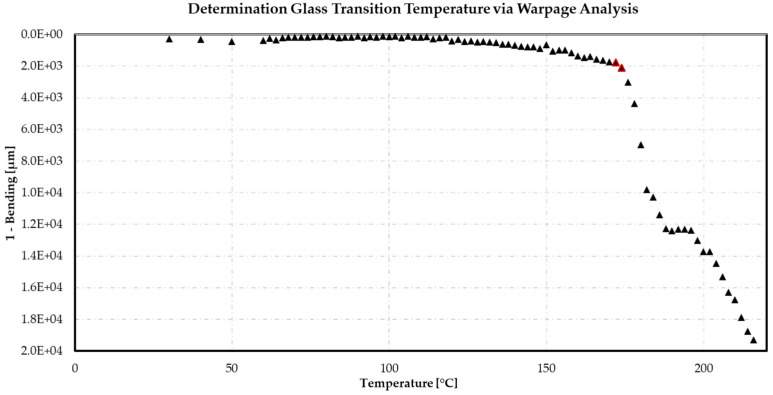
Determination of the glass transition temperature via warpage analyzer. The bending was measured during heating. The first linear curve indicates the expansion of the material and the bending caused by the heat. The subsequent significant increase in bending indicates that the *T*_g_ range has been reached. *T*_g_ was defined as the temperature between two points (marked in red) as a significant increase starts.

**Figure A8:**
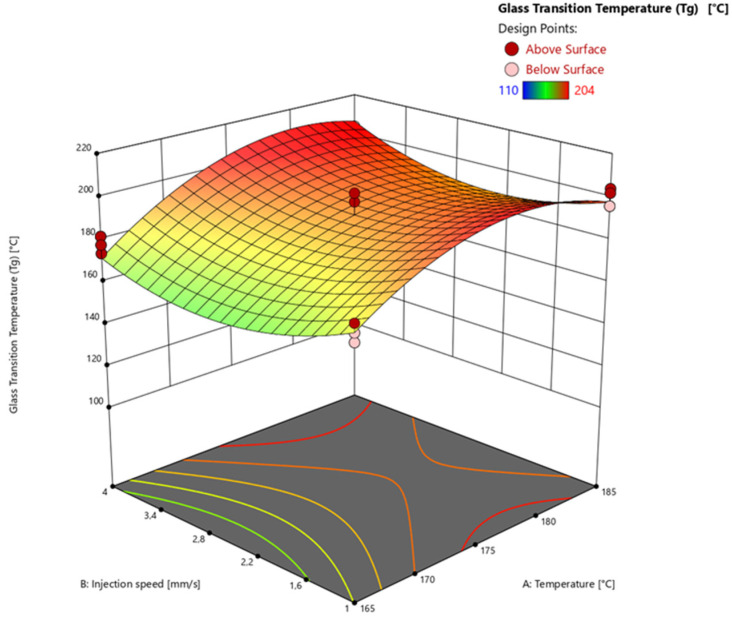
Response surface of the glass transition temperature at a heating time of 120° s is shown. Effects of transfer molding parameters temperature (A) and injection speed (B) on the glass transition temperature (*z*-axis) are plotted. Dark red and light red dots depict the measured raw values above and below the prediction surface, respectively.

The effects of the temperature and heating time on the glass transition temperature (*T*_g_) determined by DEA (*z*-axis) are shown in Figure 13 at an injection speed of 1 mm/s. An example of the warpage analysis measurement of a test point to detect the *T_g_* at a specific degree of cure is shown in Figure A5. As mentioned above, only the interactions between the important parameters of temperature and heating time are discussed for comparison purposes. The remaining response surface plots of the interaction with the injection speed are shown in the Appendix A.

Looking at the response surface shows the significant effects of temperature (A) and heating time (C) on the glass transition temperature (*T*_g_). As observed considering the response surface of the degree of cure calculated via DEA and DSC, the higher the temperature and the longer the heating time, the higher the degree of cure, resulting in a higher *T*_g_. For example, looking at axis *A* at 60 s, the *T*_g_ increases between 165 °C and 185 °C at a calculated *T*_g_ of 202 °C on average. The increase due to temperature and heating time can also be explained by kinetics of the EMC, using the Arrhenius equation, progressing the reaction and therefore the *T*_g_. Considering the C axis at 165 °C, the longer the heating time, the higher the resulting *T*_g_. The significant interaction between A and C (AC) can be seen on the middle axis of the graph. The effect on the degree of cure rises with increasing temperature and simultaneously with increasing heating time. The square significant effects A^2^ and C^2^ result comparably to the evaluation of the degree of cure from the general kinetic curve reaching a plateau towards 100% of the degree of cure. The higher effect of the temperature on the *T*_g_ compared to the heating time is evident considering axes A and C exclusively. The temperature favors the reaction and results in a higher *T*_g_. In relation to the heating time, the *T*_g_ is mainly determined by the existing temperature of the process. When *T*_g_ = *T*_processing_ is reached, the kinetic reaction stagnates and only diffusion driven crosslinking promotes further increases in *T*_g_. Overall, the curves of the degree of cure and the *T*_g_ show clearly comparable curves. The degree of cure calculated using DEA shows almost identical curves with the calculated *T*_g_, which can be explained by the previously discussed relationship between ionic viscosity and degree of cure. Consequently, DEA is a useful analytical tool for determining *T*_g_. In addition, the similarity of the cure curves determined by DSC and DEA demonstrates the applicability of the conversion calculation using the DiBenedetto equation is applicable.

## 4. Conclusions

In this work, a new method was demonstrated to determine the glass transition temperature (*T*_g_) associated with a certain degree of cure in a simple measurement principle with short cycle times. Using the DiBenedetto equation, the *λ* of the investigated material can be calculated and transferred to an implementation of the ion viscosity. The *λ* of 0.57 represents a non-linear behavior of the conversion to the glass transition temperature (*T*_g_). With regressions above 0.98, the DiBenedetto fits show a reliable representation of the data. The DiBenedetto fits were used to calculate the degree of cure associated with the *T*_g_ values and *IV*_0_ of the uncured material. The evaluation of the FCDoE showed a possible comparison between differential scanning calorimetry (DSC) and dielectric analysis (DEA) and a comparable prediction for the degree of cure as a function of process parameters. The *T*_g_ was found to correlate with the DEA. Consequently, a prediction of the *T*_g_ curve as a function of process parameters is possible by developing DEA prediction models. Process parameters such as the injection speed and temperature have an influence on the degree of cure, which is consistent with observations in previous work. An underlying shear heating due to increased injection speed with an effect on the kinetics is suspected. Since the effects were not recognized by the DEA, the sensitivity of the DEA should be reviewed. Therefore, investigations of the sensitivity of the DEA sensors in dependence on the filler, e.g., silica particle content, could provide more detailed information on possible inhomogeneity phenomena. Based on the relationship between DEA and *T*_g_, the *T*_g_ of the material can be monitored during the molding process. The use of DiBenedetto for ionic viscosity allows the calculation of *T*_g_ values in crosslinking ranges that are difficult to obtain from thermomechanical measurements. In addition, the evolution of ionic viscosity during processing can represent the evolution of *T*_g_ independent of fillers and can also determine the achievement of *T*_g_ under certain conditions during production. However, it should be emphasized that the degree of cure $\alpha_{IV}$ measured by the DEA cannot be compared to the DSC ($\alpha_{H})$ without a validation of the DiBenedetto application by calculating the ion viscosity of the uncured material (*IV*_0_) The applicability of DEA to determine the degree of cure and *T*_g_ of polymers, as presented in this work, can be extended to other polymers as long as ionic viscosity is measurable and affected by crosslinking. The new method presented for calculating *T*_g_ provides a fast and simple method for determining *T*_g_ at a given level of crosslinking. In addition, investigations into the resolution of the warpage measurement method can verify the applicability of the method to larger samples, such as finished components.

## Figures and Tables

**Table 1:** Factor level settings and corresponding values of responses evaluated in the design of experiment (DoE).

	Factor 1	Factor 2	Factor 3	Response 1	Response 2	Response 3
Run	A: Temperature [°C]	B: Injection speed [mm/s]	C: Heating time [s]	Degree of Cure via DEA [%]	Degree of Cure via DSC [%]	Glass Transition Temperature (*Tg*) [°C]
1	165	1	60	75.08	74.21	144
2	165	1	60	75.43	72.51	138
3	165	1	60	74.34	71.77	144
4	185	1	60	95.38	97.67	202
5	185	1	60	97.08	96.12	202
6	185	1	60	96.75	97.04	196
7	165	4	60	74.22	74.99	114
8	165	4	60	73.65	72.31	114
9	165	4	60	75.83	78.27	110
10	185	4	60	97.87	100.00	196
11	185	4	60	97.47	100.00	200
12	185	4	60	98.11	100.00	202
13	165	1	120	95.56	92.37	174
14	165	1	120	96.56	92.72	182
15	165	1	120	96.11	93.90	178
16	185	1	120	98.55	100.00	204
17	185	1	120	98.53	100.00	196
18	185	1	120	98.30	100.00	202
19	165	4	120	95.34	93.58	174
20	165	4	120	95.58	93.25	182
21	165	4	120	95.40	93.47	178
22	185	4	120	98.29	100.00	200
23	185	4	120	98.34	100.00	202
24	185	4	120	98.06	100.00	204
25	175	2.5	90	97.55	96.67	190
26	175	2.5	90	97.69	97.64	188
27	175	2.5	90	97.66	97.49	192
28	175	2.5	90	97.55	94.83	192
29	175	2.5	90	97.18	96.83	182
30	165	2.5	90	86.69	86.54	136
31	165	2.5	90	86.83	87.09	128
32	165	2.5	90	86.49	86.86	134
33	185	2.5	90	98.55	100.00	202
34	185	2.5	90	98.38	100.00	196
35	185	2.5	90	98.54	100.00	202
36	175	1	90	97.23	96.92	196
37	175	1	90	97.17	100.00	200
38	175	1	90	97.29	96.94	198
39	175	4	90	96.42	100.00	190
40	175	4	90	96.56	96.96	196
41	175	4	90	96.77	96.48	196
42	175	2.5	60	91.20	94.91	150
43	175	2.5	60	88.99	94.28	146
44	175	2.5	60	90.43	95.43	158
45	175	2.5	120	97.72	98.54	198
46	175	2.5	120	97.76	98.45	202
47	175	2.5	120	97.88	98.70	190

**Table 2:** Analysis of variances and regressions of the gained response surface models the FCDoE of the response degree of cure calculated via DEA.

Source	Sum of Squares	df	Mean Square	F-Value	*p*-Value
Block	42.72	1	42.72		
Model	2748.42	5	549.68	741.31	<0.0001
A-Temperature	1141.70	1	1141.70	1539.71	<0.0001
C-Heating time	812.67	1	812.67	1095.98	<0.0001
AC	586.13	1	586.13	790.46	<0.0001
A^2^	111.87	1	111.87	150.87	<0.0001
C^2^	44.59	1	44.59	60.13	<0.0001
Residual	29.66	40	0.7415		
Lack of Fit	21.22	8	2.65	10.05	<0.0001
Pure Error	8.44	32	0.2639		

**Table 3:** Analysis of variances and regressions of the gained response surface models the FCDoE of the response degree of cure calculated via DSC.

Source	Sum of Squares	df	Mean Square	F-Value	*p*-Value
Block	149.84	1	149.84		
Model	2969.13	4	742.28	226.44	<0.0001
A-Temperature	1717.32	1	1717.32	523.87	<0.0001
C-Heating time	611.78	1	611.78	186.63	<0.0001
AC	468.66	1	468.66	142.97	<0.0001
A^2^	171.37	1	171.37	52.28	<0.0001
Residual	134.40	41	3.28		
Lack of Fit	91.43	9	10.16	7.57	<0.0001
Pure Error	42.97	32	1.34		

**Table 4:** Analysis of variances and regressions of the gained response surface models the FCDoE of the response glass transition temperature.

Source	Sum of Squares	df	Mean Square	F-Value	*p*-Value
Block	0.0862	1	0.0862		
Model	34,913.18	9	3879.24	83.02	<0.0001
A-Temperature	20,072.53	1	20,072.53	429.58	<0.0001
B-Injection speed	320.13	1	320.13	6.85	0.0129
C-Heating time	6750.00	1	6750.00	144.46	<0.0001
AB	337.50	1	337.50	7.22	0.0108
AC	3601.50	1	3601.50	77.08	<0.0001
BC	368.17	1	368.17	7.88	0.0080
A^2^	1977.34	1	1977.34	42.32	<0.0001
B^2^	1253.40	1	1253.40	26.82	<0.0001
C^2^	567.71	1	567.71	12.15	0.0013
Residual	1682.13	36	46.73		
Lack of Fit	1189.33	4	297.33	19.31	<0.0001
Pure Error	492.80	32	15.40		

**Figure A6:**
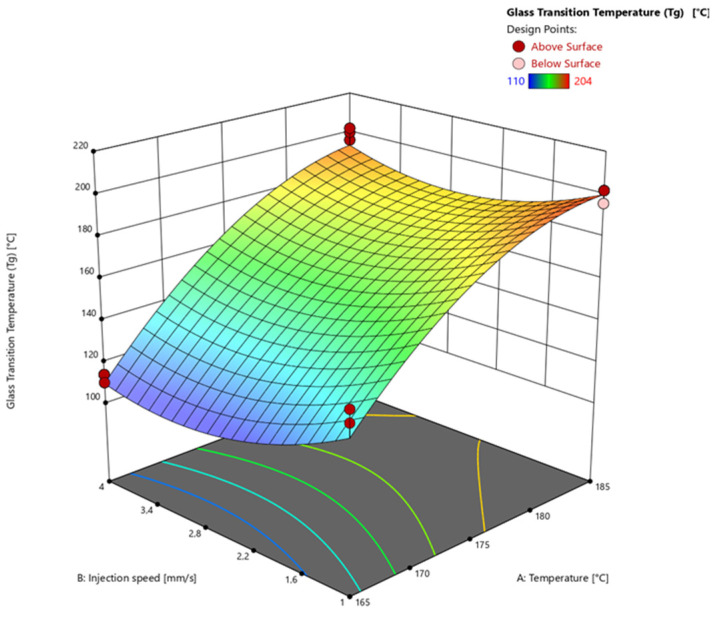
Response surface of the glass transition temperature at a heating time of 60° s is shown. Effects of transfer molding parameters temperature (A) and injection speed (B) on the glass transition temperature (*z*-axis) are plotted. Dark red and light red dots depict the measured raw values above and below the prediction surface, respectively.

**Figure A7:**
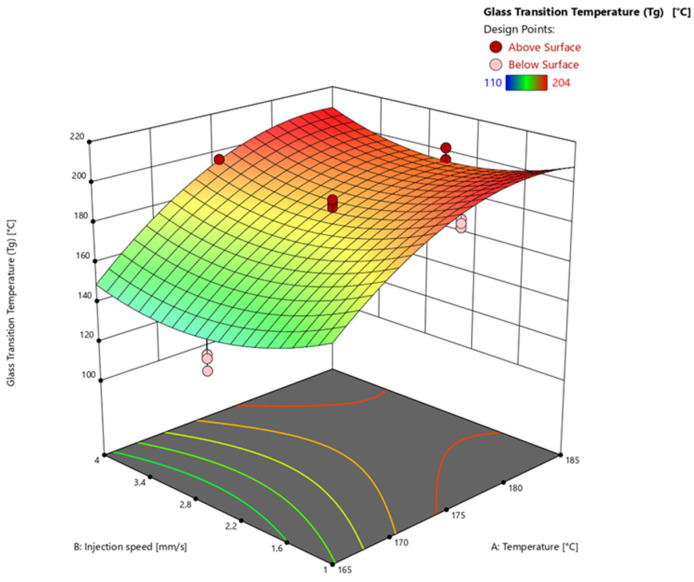
Response surface of the glass transition temperature at a heating time of 90° s is shown. Effects of transfer molding parameters temperature (A) and injection speed (B) on the glass transition temperature (*z*-axis) are plotted. Dark red and light red dots depict the measured raw values above and below the prediction surface, respectively.

**Figure A9:**
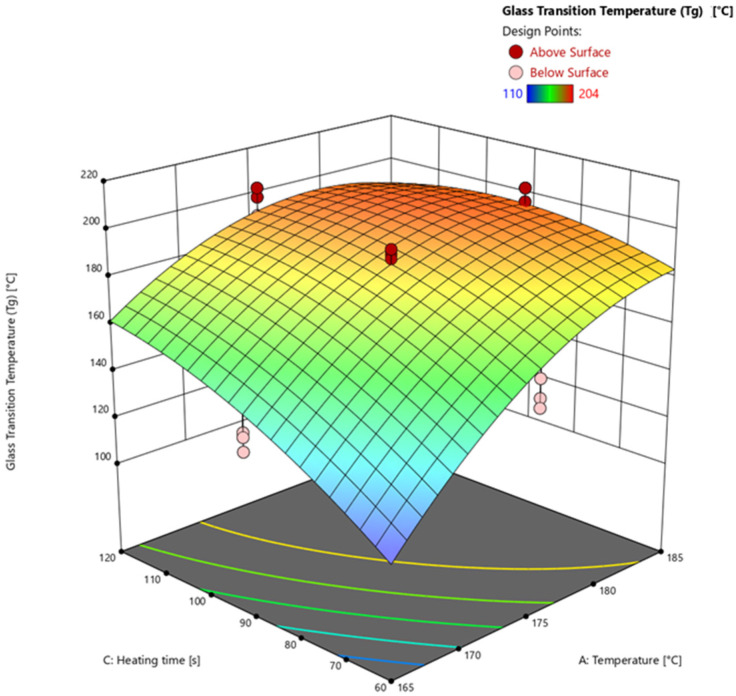
Response surface of the glass transition temperature calculated via warpage analysis shown at an injection speed of 2.5 mm/s. Effects of transfer molding parameters temperature (A) and heating time (C) on the degree of cure (*z*-axis) are plotted. Dark red and light red dots depict the measured raw values above and below the prediction surface, respectively.

**Figure A10:**
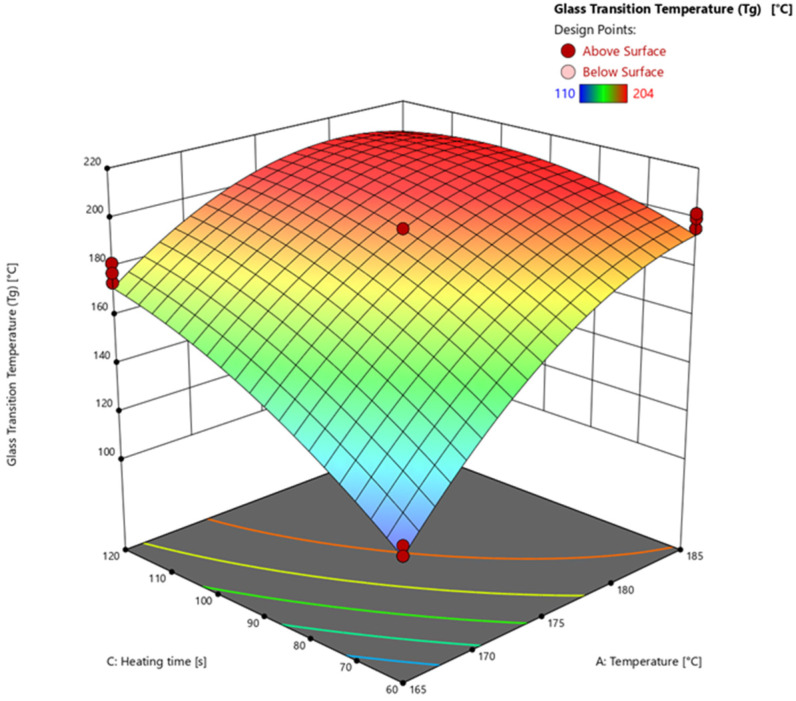
Response surface of the glass transition temperature calculated via warpage analysis shown at an injection speed of 4 mm/s. Effects of transfer molding parameters temperature (A) and heating time (C) on the degree of cure (*z*-axis) are plotted. Dark red and light red dots depict the measured raw values above and below the prediction surface, respectively.

**Figure A11:**
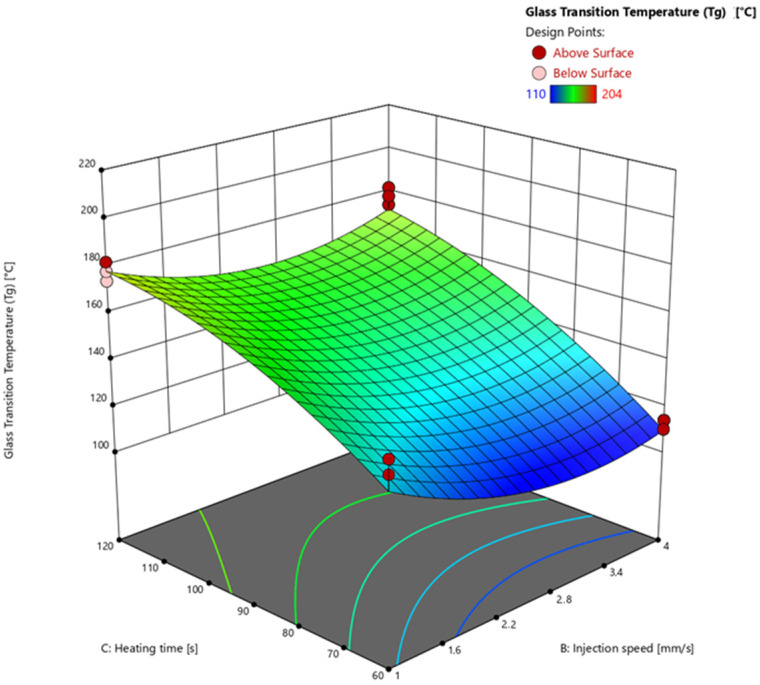
Response surface of the glass transition temperature calculated via warpage analysis shown at a temperature of 165 °C. Effects of transfer molding parameters injection speed (B) and heating time (C) on the degree of cure (*z*-axis) are plotted. Dark red and light red dots depict the measured raw values above and below the prediction surface, respectively.

**Figure A12:**
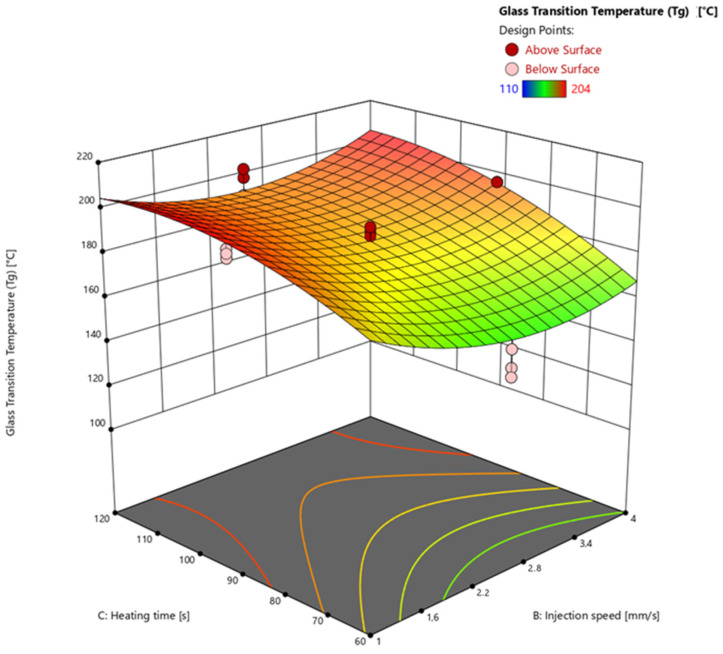
Response surface of the glass transition temperature calculated via warpage analysis shown at a temperature of 175 °C. Effects of transfer molding parameters injection speed (B) and heating time (C) on the degree of cure (*z*-axis) are plotted. Dark red and light red dots depict the measured raw values above and below the prediction surface, respectively.

**Figure A13:**
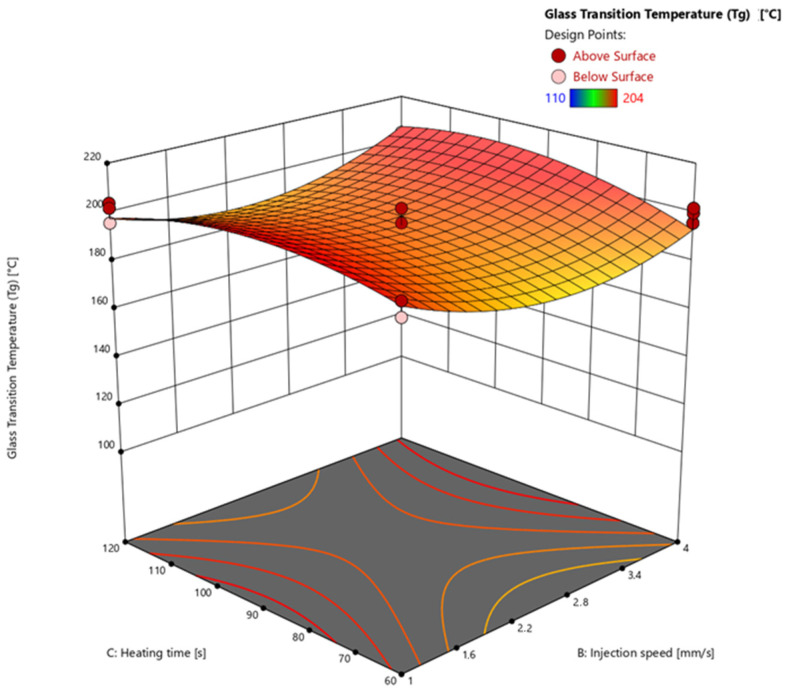
Response surface of the glass transition temperature calculated via warpage analysis shown at a temperature of 185 °C. Effects of transfer molding parameters injection speed (B) and heating time (C) on the degree of cure (*z*-axis) are plotted. Dark red and light red dots depict the measured raw values above and below the prediction surface, respectively.

**Figure A14:**
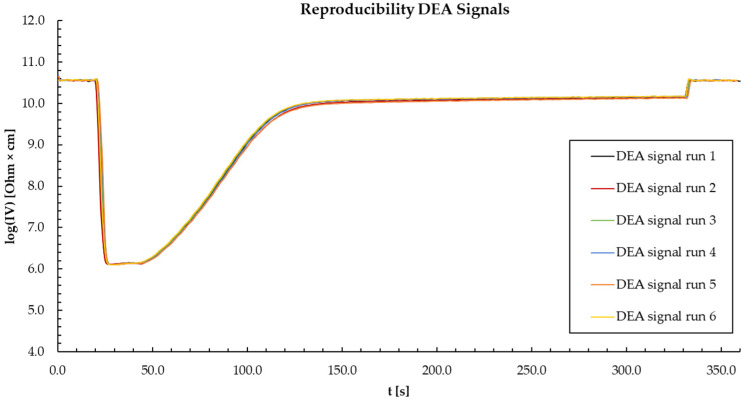
An example of raw data of DEA measurements of EMC at a temperature of 175 °C and an injection speed of 2.5 mm/s at a heating time of 300 s reaching a plateau. Shown are the raw signals of the six repeated measurements on sensor 1.

## Data Availability

Data are available upon request from the authors.
